# Effect of early administration of dexamethasone in patients with COVID-19 pneumonia without acute hypoxemic respiratory failure and risk of development of acute respiratory distress syndrome: EARLY-DEX COVID-19 trial

**DOI:** 10.3389/fmed.2024.1385833

**Published:** 2024-07-17

**Authors:** Anabel Franco-Moreno, María Soledad Acedo-Gutiérrez, Miguel Ángel Casado-Suela, Nicolás Labrador-San Martín, María de Carranza-López, Fátima Ibáñez-Estéllez, Clara Hernández-Blanco, José Jiménez-Torres, Ignacio Vallejo-Maroto, Rodolfo Romero-Pareja, Gabriela Peña-Lillo, Ismael Escobar-Rodríguez, Juan Torres-Macho, EARLY-DEX COVID-19 Research Group, Belén Escolano-Fernández

**Affiliations:** ^1^Internal Medicine Department, Hospital Universitario Infanta Leonor–Virgen de la Torre, Madrid, Spain; ^2^Internal Medicine Department, Hospital Universitario Puerta de Hierro, Madrid, Spain; ^3^Internal Medicine Department, Hospital Carlos III, Madrid, Spain; ^4^Hospital Emergencia COVID-19, Sevilla, Spain; ^5^Hospital de Emergencias Enfermera Isabel Zendal, Madrid, Spain; ^6^European University, Madrid, Spain; ^7^Emergency Department, Hospital Universitario Infanta Leonor–Virgen de la Torre, Madrid, Spain; ^8^Pharmacology Department, Hospital Universitario Infanta Leonor–Virgen de la Torre, Madrid, Spain; ^9^Department of Medicine, Complutense University, Madrid, Spain

**Keywords:** acute respiratory distress syndrome, biomarkers, COVID-19 management, dexamethasone, inflammation, mortality, pneumonia, randomized controlled trial

## Abstract

**Introduction and objectives:**

Corticosteroids are among the drugs demonstrating a mortality benefit for coronavirus disease 2019 (COVID-19). The RECOVERY trial highlighted that dexamethasone reduced 28-day mortality for hospitalized COVID-19 patients requiring either supplemental oxygen or mechanical ventilation. It is noted that approximately 30% of COVID-19 patients, initially presenting with mild symptoms, will advance to acute respiratory distress syndrome (ARDS), especially those with detectable laboratory markers of inflammation indicative of disease progression. Our research aimed to explore the efficacy of dexamethasone in preventing the progression to ARDS in patients hospitalized with COVID-19 pneumonia who do not yet require additional oxygen but are at high risk of developing ARDS, potentially leading to a reduction in morbimortality.

**Methods:**

In this multicenter, randomized, controlled trial, we evaluated the impact of dexamethasone on adult patients diagnosed with COVID-19 pneumonia who did not need supplementary oxygen at admission but were identified as having risk factors for ARDS. The risk of ARDS was determined based on specific criteria: elevated lactate dehydrogenase levels over 245 U/L, C-reactive protein levels exceeding 100 mg/L, and a lymphocyte count below 0.80 × 10^9^/L. Participants were randomly allocated to either receive dexamethasone or the standard care. The primary endpoints included the incidence of moderate or severe ARDS and all-cause mortality within 30 days post-enrollment.

**Results:**

One hundred twenty-six patients were randomized. Among them, 41 were female (30.8%), with a mean age of 48.8 ± 14.4 years. Ten patients in the dexamethasone group (17.2%) and ten patients in the control group (14.7%) developed moderate ARDS with no significant differences. Mechanical ventilation was required in six patients (4.7%), with four in the treatment group and two in the control group. There were no deaths during hospitalization or during follow-up. An intermediate analysis for futility showed some differences between the control and treatment groups (Z = 0.0284). However, these findings were within the margins close to the region where the null hypothesis would not be rejected.

**Conclusion:**

In patients with COVID-19 pneumonia without oxygen needs but at risk of progressing to severe disease, early dexamethasone administration did not lead to a decrease in ARDS development.

**Clinical trial registration:**

ClinicalTrials.gov, identifier NCT04836780.

## Introduction

Corticosteroids are the cornerstone of treatment in hospitalized patients with coronavirus disease (COVID-19) and respiratory failure. Its administration has been associated with clinical advantages, including a reduction in mortality rates. This infection can precipitate a cytokine storm, marked by an exaggerated inflammatory reaction to SARS-CoV-2, driven by an unbalanced immune response from the host (1). Prompt intervention in the cytokine storm is crucial to avert the progression to critical, potentially fatal conditions, predominantly due to acute respiratory distress syndrome (ARDS) (2, 3).

Current COVID-19 Treatment Guidelines recommend dexamethasone in patients who require respiratory support (4–6). In the Randomized Evaluation of COVid-19 thERapY (RECOVERY) trial, daily use of 6 mg dexamethasone for ten days resulted in lower 28-day mortality in patients hospitalized with COVID-19 who were receiving supplemental oxygen or invasive mechanical ventilation, but not among those receiving no respiratory support at randomization (7). Similarly, in a prospective meta-analysis that pooled data from seven randomized clinical trials of critically ill patients with COVID-19, systemic corticosteroid administration was associated with lower 28-day all-cause mortality compared to usual care or placebo (8).

Previous studies have shown that 30% of patients with COVID-19 could progress to severe disease, mainly due to ARDS development (1). Specific laboratory parameters, such as inflammatory biomarkers, are related to progression to ARDS and mortality in COVID-19 patients. Available data provide evidence for the differentiation of severe and non-severe cases of COVID-19 based on these biomarkers, including lymphocyte count, lactate dehydrogenase (LDH) and C-reactive protein (CRP) (9–12). In patients with COVID-19, elevated LDH increases mortality (13). For each 100-unit increase in CRP levels, the odds of death increase two-fold (14). Finally, a lymphocyte count lower than 0.95 × 10^9^/L is associated with a risk of death compared to patients with lymphocyte counts greater than 0.95 × 10^9^/L (15). Therefore, these parameters can help to identify patients at high risk of ARDS and death who might potentially benefit from early treatment with corticosteroids. Depending on the degree of underlying inflammation, corticosteroids might have a different effect in patients with COVID-19 in its initial stage. In this regard, the RECOVERY trial did not differentiate among patients with and without elevated inflammatory parameters who did not require oxygen.

We postulated that dexamethasone in patients at high risk of ARDS development based on inflammatory biomarkers of disease progression could help to control cytokine storm and reduce mortality.

The EARLY-DEX COVID-19 trial evaluated the efficacy of the early administration of corticosteroid treatment in hospitalized patients with COVID-19 pneumonia without additional oxygen requirements on admission, in whom biomarkers showed a high risk of ARDS development.

## Materials and methods

### Trial design

The effect of early administration of dexamethasone in patients with COVID-19 pneumonia without acute hypoxemic respiratory failure and risk of development of acute respiratory distress syndrome (EARLY-DEX COVID-19) study is a prospective multicenter, randomized, controlled, open-label, parallel-group trial. The trial protocol and statistical analysis plan have been published previously (16).

The trial was designed according to the Declaration of Helsinki (17) and the Convention of the European Council related to human rights and biomedicine and complied with the requirements established by Spanish legislation in biomedical research, the protection of personal data, and bioethics. It was registered on April 8, 2021, at http://www.clinicaltrials.gov with identification no. NCT04836780. The Ethics Committee approved the study protocol (Version 1.2, April 17, 2021) for the investigation of medicinal products of the Comunidad de Madrid, Spain, and the institutional review boards of all participating hospitals. The Spanish Agency for Drugs and Medical Devices approved the trial as a clinical randomized drug study on May 25, 2021.

Informed consent was obtained from the patients or their legal surrogates according to national regulations. Following the recommendations of the Spanish Agency of Medicines and Medical Devices, during the COVID-19 pandemic, patient consent could be obtained orally and was to be obtained preferably before an impartial witness, with documentation in the patient’s medical records (18).

### Trial sites

Patients were randomized and enrolled in Spain. Study sites included Hospital de Emergencias Enfermera Isabel Zendal, a specialized center for the treatment of SARS-CoV-2 infection; Hospital Universitario Infanta Leonor–Virgen de la Torre, both in Madrid; and Hospital de Emergencia COVID-19 in Sevilla. The study was conducted during Spain’s third and fourth COVID-19 waves, with roughly half of the sample collected per wave. During this period, the proportion of adults 70 and older vaccinated against COVID-19 reached 80% (19).

### Patients

Eligible patients were aged 18 years or older, hospitalized due to COVID-19 pneumonia, did not require supplemental oxygen on admission, and were at risk of developing ARDS, defined by the presence of at least two of the following inflammatory biomarkers: LDH >245 units/L, CRP >100 mg/L, or lymphocyte count <0.80 × 10^9^/L (Table 1).

**Table 1:** Inclusion criteria.

Adults (age 18 years or older)
Confirmed COVID-19 based on a positive RT-PCR test or rapid antigen test for SARS-CoV-2 RNA in a respiratory specimen (nasopharyngeal or nasal swab)
Requiring in-hospital care
A chest imaging study compatible with pneumonia (X-ray or computed tomography)
SpO2 ≥ 94 per cent and < 22 bpm breathing on room air
The presence of at least two of the following inflammatory biomarkers: LDH >245 U/L CRP >100 mg/L Lymphocyte count <0.80 × 10^9^/L

SpO2, peripheral capillary oxygen saturation; bpm, breaths per minute; LDH, lactate dehydrogenase; CRP, C-reactive protein.

### Randomization

Eligible, consenting patients were randomly assigned in a 1:1 ratio to receive either dexamethasone plus standard of care (intervention group) or standard of care alone (control group). Randomization was performed using the REDCap system. A randomization schedule using a permuted block design was created, ensuring equal numbers were assigned to each group. Records of individual assignments to each group were anonymized (user number and centre ID instead of patients’ names), sequentially numbered, and stored on a secure computer system. Local investigators in participating centers were the only authorized personnel to interact with the randomization system through a username and password. Once eligibility was confirmed in the electronic case report form, the system assigned the treatment group. Dexamethasone was provided free of charge for this study by the funding organization. In line with the ethical principles for medical research outlined in the Declaration of Helsinki, no placebo was considered appropriate when an effective intervention exists. The Spanish Agency of Drugs and Medical Devices and the Ethics Committees did not require a blinded design or the administration of a placebo.

### Intervention

Patients in the intervention group received 6 mg of dexamethasone once daily for seven days. Participants unable to take oral drugs received intravenous dexamethasone. Participants randomized to the control group received standard of care. Given that pulmonary disease can progress rapidly in COVID-19, patients were closely monitored. Following practice guidelines, corticosteroids were administered to patients in the control group who required oxygen support. Remdesivir, Tocilizumab, Baricitinib, Tofacitinib, and Sarilumab were considered in cases of worsening disease status at the attending physician’s discretion (4–6). At the discretion of the responsible physician, all patients could receive low-molecular-weight heparin to prevent venous thromboembolic disease, prophylaxis for gastric ulcers, hydration, antipyretics, antibiotics, and bronchodilators.

Investigators had the authority to modify or discontinue the assigned study intervention if serious adverse events occurred during the study period or if participant consent was withdrawn. Regardless of any decision to discontinue their assigned intervention, study participants were retained in the trial whenever possible to enable follow-up data collection and to minimize missing data.

### Data collection

The trial investigators or staff reported any serious adverse events to the coordinating centers and entered the baseline characteristics, process variables, and outcome data from the patient files into web-based case report forms. Trial data were monitored at the sites according to a predefined monitoring plan.

### Outcomes

The primary outcome was a composite of moderate or more severe ARDS development and all-cause 30 days mortality after enrollment. According to the Berlin criteria, moderate ARDS was defined by the partial pressure of arterial oxygen/fraction of inspired oxygen (PaO2/FiO2) >100 mmHg and ≤ 200 mmHg, and severe ARDS by a PaO2/FiO2 < 100 mmHg (20). The PaO2/FiO2 ratio was monitored at least once daily to determine ARDS development. Site investigators reported patient status at 30 days, regardless of whether the patient was still hospitalized or had been discharged. Readmissions with moderate ARDS and mortality at 30 days after randomization were detected on follow-up telephone calls or during electronic medical record review sessions.

Secondary outcomes were requirement of non-invasive or invasive mechanical ventilation, hospital length of stay, and 90-days all-cause mortality after enrollment.

### Evaluation of the radiographic severity of COVID-19 pneumonia

The Radiographic Assessment of Lung Edema (RALE) score was applied to assess COVID-19 pneumonitis extension. RALE score divides each hemithorax into four quadrants. To calculate the RALE score, each quadrant scores one. Therefore, the minimum RALE score is 0 (no infiltrates), and the maximum RALE score is 8 (consolidation in all quadrants).

### Sample size calculation

Based on available evidence, we estimated a 30% risk of ARDS in the control group (1). We aimed for an absolute 10% reduction in the intervention group. Based on an assumption of 80% statistical power and a two-sided significance level (alpha) of 0.05, it was estimated that a total of 226 participants were required. We opted for a sample size of 252 patients (126 per group) to compensate for the potential loss of some participants to follow-up.

### Statistical analysis

Mean and standard deviation were used to define quantitative variables. Continuous variables were compared using the Student’s t-test (normal distribution) or Mann–Whitney test (non-normal distribution). Qualitative variables were defined by frequency and compared using the Chi-square or Fisher’s exact test. The primary endpoint was analyzed using the Chi-squared test. The log-rank test evaluated Patient survival using Kaplan–Meier analysis, and groups were compared. A *p*-value of less than 0.05 was considered statistically significant. Statistical analysis was performed with SPSS v28.0 software (SPSS, Inc., Chicago, Illinois, United States). Due to the end of the COVID-19 surge and the lower-than-expected incidence of ARDS during study recruitment, an intermediate futility analysis was performed.

## Results

### Participants

**Figure 1:**
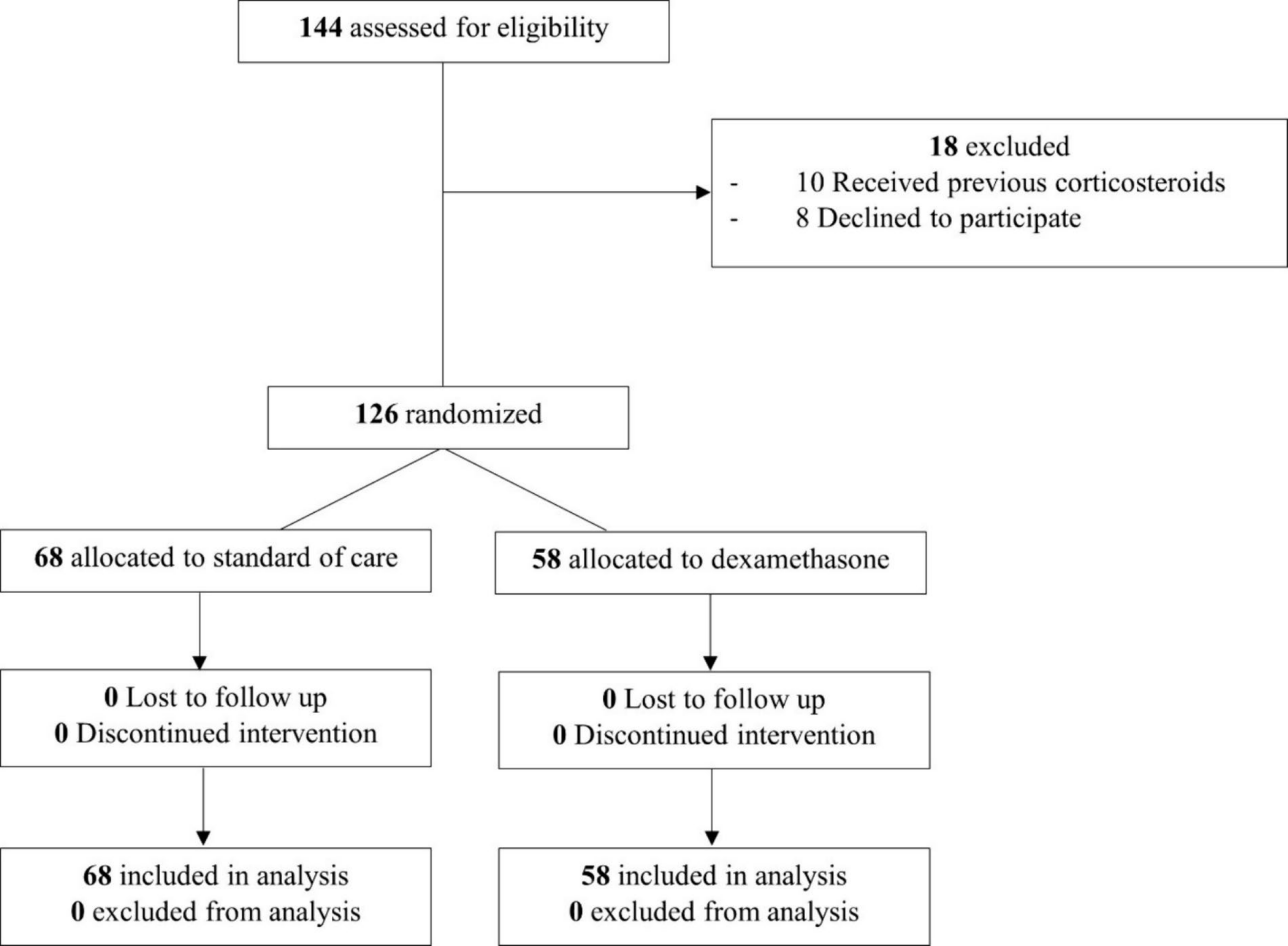
Study flowchart.

Between June 2021 and January 2022, 144 patients were evaluated for eligibility. Eighteen patients were excluded, resulting in 126 patients being randomized for the study, all of whom were included in the final analysis, as depicted in the study flowchart; 58 were assigned to the dexamethasone group and 68 to the control group (Figure 1). Among these, 41 were female (30.8%), with an average age of 48.8 ± 14.4 years. No statistically significant differences were observed between groups. It is important to note that hypertension, diabetes, dyslipidemia, and obesity were the most common comorbidities, however a low proportion of patients showed chronic obstructive pulmonary disease (COPD), asthma, heart failure, or chronic renal failure. Approximately one-third of the participants had received the SARS-CoV-2 vaccination. The median duration from the onset of symptoms to study inclusion was nine days (interquartile range: 8–10 days). During the study, one patient received Remdesivir (1.5% of the 68 patients in the control group). Twelve and 13 patients received antibiotics, four and two received Tocilizumab, and two and three received hyperimmune plasma in the dexamethasone and control groups, respectively. No patients received Baricitinib, Tofacitinib, or Sarilumab. According to the RALE score, there were no significant differences between pneumonitis extension among patients who developed ARDS and those who did not. Demographic and clinical characteristics, treatments and outcomes of both groups are detailed in Table 2.

**Table 2:** Patient clinical profiles in the study.

	Total *n* = 126	Control group *n* = 68	Intervention group *n* = 58	*p*
**Demographics data**				
Age (mean)	48.8 (14.5)	49.3 (14.3)	48.2 (14.8)	0.68
Female gender	41 (32.5)	23 (33.8)	18 (31)	0.80
BMI	28 (4.9)	28.5 (5)	27.4 (4.8)	0.25
**Previous conditions**				
Hypertension	29 (23)	15 (22)	15 (24.1)	0.80
Dyslipiedemia	20 (15.8)	9 (13.2)	11 (18.9)	0.46
Diabetes Mellitus	19 (15)	8 (11.7)	11 (18.9)	0.31
Obesity	36 (28.1)	21 (30.8)	15 (25)	0.57
Smoker	4 (3.1)	1 (1.4)	3 (5.1)	0.33
Cerebrovascular disease	1 (3.1)	0	1 (1.6)	0.45
Coronary heart disease	2 (1.5)	1 (1.4)	1 (1.7)	0.70
Active neoplasm	1 (0.78)	0	1 (1.7)	0.45
OSA	4 (3.1)	3 (4.4)	1 (1.7)	0.62
Chronic renal failure	3 (2.3)	2 (2.9)	1 (1.7)	0.56
Asthma	4 (3.1)	3 (4.4)	1 (1.7)	0.62
Previous vaccination	37 (29.3)	24 (35.2)	13 (22.4)	0.12
**Clinical characteristics at admission**				
Days to inclusion (mean)	9 (3.2)	9 (3.5)	8.6 (2.9)	0.56
Temperature	36.9 (0.75)	37 (0.79)	36.9 (0.69)	0.59
Heart beats per minute	81.9 (12)	82.9 (12.3)	80.8 (12.4)	0.34
Respiratory frequency	18 (2.7)	17.7 (3.1)	18.6 (2.2)	0.052
Systolic blood pressure	119 (18)	118.8 (17)	119.6 (20.2)	0.81
O2 saturation (ambient air)	95.8 (1.18)	96 (1.2)	95.6 (1.1)	0.13
PaO2/FiO2	395 (61.2)	400.8 (73.6)	389.1 (41.5)	0.29
**Analytical and radiological parameters at admission**				
Haemoglobin	14.1 (1.4)	14 (1.5)	14.2 (1.4)	0.43
Limphocytes	1,070 (841)	1106.8 (706)	1,027 (984)	0.60
Glucose	107.2 (27.5)	104.5 (23.7)	110.3 (31.3)	0.24
Creatinine	0.8 (0.22)	0.83 (0.26)	0.78 (0.18)	0.23
Albumin	4 (0.82)	4.04 (0.81)	4.07 (0.85)	0.9
Sodium	137 (2.7)	137.3 (2.4)	137.5 (39)	0.71
LDH	331 (107)	327.8 (105)	334.9 (111)	0.71
C-reactive protein	98 (71.9)	96.7 (76.3)	99.7 (66.9)	0.81
D-dimer	794.2 (1230.3)	889.7 (1629)	684 (456)	0.40
Ferritin	677.5 (560)	673.8 (526)	682 (605)	0.94
Unilateral infiltrate	14 (11)	10 (14.7)	4 (6.8)	0.25
Number of affected areas	3.1 (1.7)	3.1 (1.7)	3.2 (1.8)	0.65
**RALE score**	4	4	4	0.18
**Medication use**				
Antibiotic	25 (19.8)	13 (19.1)	12 (20.6)	0.86
LMWH	126 (100)	68 (100)	58 (100)	1.0
Remdesivir	1 (1.5)	1 (1.5)	0	1.0
Tocilizumab	6 (4.7)	2 (2.9)	4 (6.8)	0.41
Hyperimmune plasma	5 (3.9)	3 (4.4)	2 (3.4)	0.62
**Primary and secondary endpoints**				
NIMV	6 (7.8)	2 (2.9)	4 (9.6)	0.71
ICU admission	1 (0.7)	0 (0)	1 (1.6)	0.45
Moderate–severe ARDS	20 (15.8)	10 (14.7)	10 (17.2)	0.8
Death	0	0	0	—
Lenght of stay (days)	6.5 (48.6)	6.6 (8.7)	6.4 (5)	0.89
**Complications**				
Infections	20 (15.6)	11 (16.1)	9 (15)	0.59
Hyperglucemia	5 (3.9)	3 (4.4)	2 (3.3)	0.6

ARDS, to acute respiratory distress syndrome; BMI, body mass index; ICU, intensive care unit; LDH, lactate dehydrogenase; LMWH, low-molecular-weight heparin; NIMV, non-invasive mechanical ventilation; OSA, obstructive sleep apnea; PaO2/FiO2, partial pressure of arterial oxygen/fraction of inspired oxygen.

### Primary outcome

**Figure 2:**
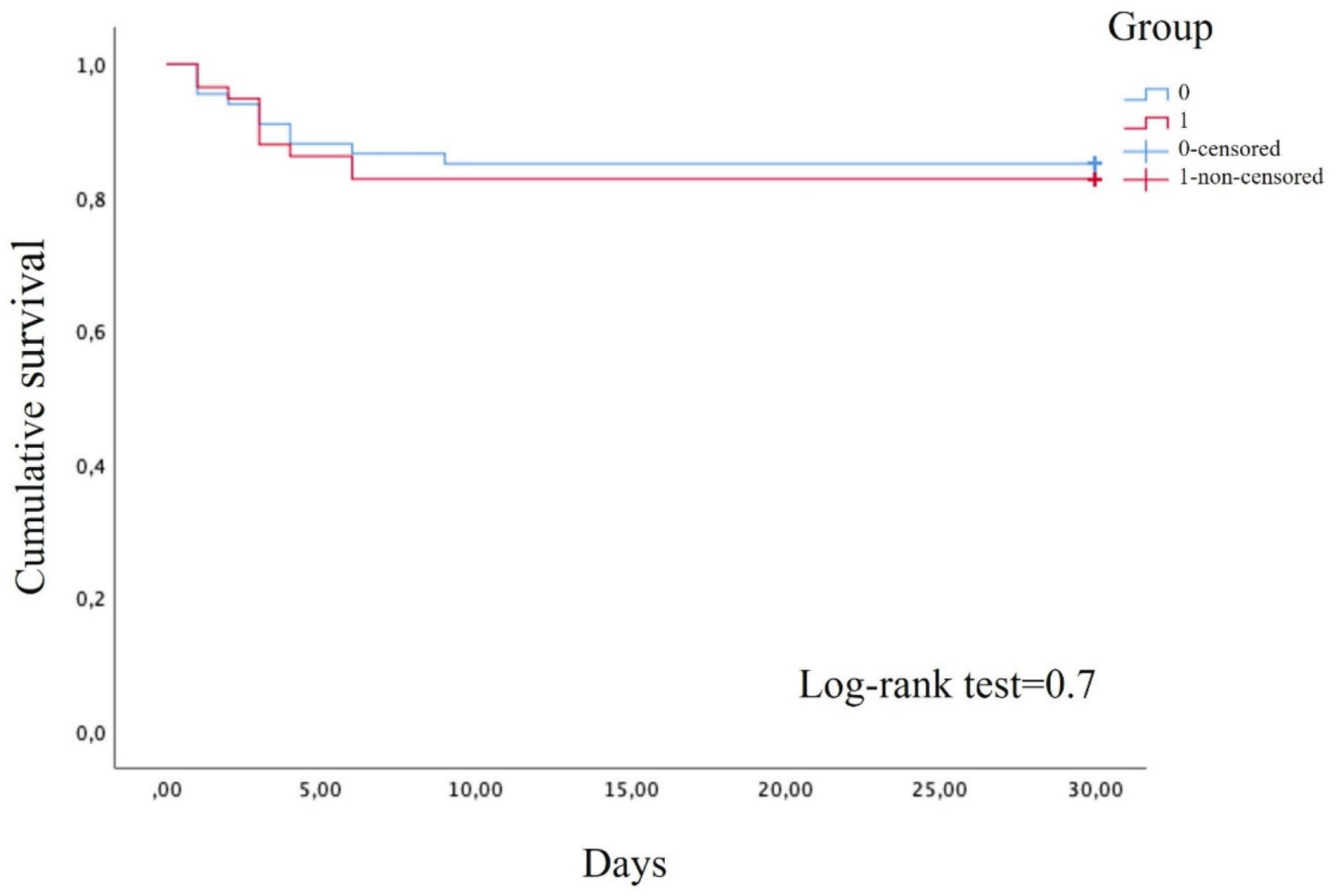
Survival analysis by log-rank test.

Throughout the study period, moderate ARDS developed in ten patients in the control group (14.7%) and ten in the treatment group (17.2%), with no statistically significant differences between the groups, as shown in the survival analysis (Figure 2).

### Secondary outcomes

Mechanical ventilation, either noninvasive or invasive, was required for six patients (4.7%), with two in the control group and four in the treatment group showing no significant differences. The average hospital length of stay was 8 ± 8.4 days, with no significant differences between the groups. There were no deaths during hospitalization or in the follow-up period.

### Adverse events

Six patients experienced moderate hyperglycemia (one in the control group and five in the treatment group), with no significant differences observed. Twenty infections were reported (15.8%), with eleven in the control group (16.1%) and nine in the intervention group (15.1%), showing no significant differences (*p* = 0.57). These included eight cases of pneumonia, three urinary tract infections, eight instances of bacteremia, and one soft tissue infection. All adverse events were considered as non-serious by the attending physicians.

### Intermediate analysis

An intermediate analysis for futility indicated some differences between the control and treatment groups (Z = 0.0284). However, these findings were within the margins close to the region where the null hypothesis would not be rejected (Z > 0.04888 and < 0.024658), as detailed in Supplementary material.

## Discussion

The main finding of our study is that early dexamethasone administration in patients with SARS-CoV-2 pneumonia at high risk of ARDS development does not substantiate a clear benefit in preventing ARDS.

Although different studies have analyzed the role of corticosteroids in patients with COVID-19 without the need for oxygen support, the primary evidence comes from the RECOVERY trial, which demonstrated its benefits in patients who require oxygen. The study mentions in its conclusions the potential harm of using steroids in patients not requiring any oxygen support. In the group of 1.534 patients with no oxygen requirements, the 28-day mortality was greater in the dexamethasone arm (18% versus 14%) (7).

In addition to the RECOVERY trial, a small sample size randomized controlled trial conducted by Tang et al. did not find benefit in the early administration of methylprednisolone (1 mg/kg daily) in patients with COVID-19 pneumonia without respiratory failure (21).

Despite these results and clinical practice guidelines recommendations (22), there is evidence showing that patients with mild COVID-19 without respiratory insufficiency but at high risk of clinical deterioration are frequently treated using corticosteroids (23).

We noted that both trials only used oxygen saturation levels as inclusion criteria (7, 21). Setting individualized targets based not only on oxygen saturation levels but also on other risk markers of disease progression to severe illness, like inflammatory biomarkers, could improve the selection of patients who may benefit from early corticosteroid administration.

The EARLY-DEX trial targeted this specific patient subset. This stratification may be critical because it identifies a population with a hyperactive immune response where the immunomodulatory effects of corticosteroids could be beneficial in preventing respiratory deterioration. This approach is based on the hypothesis that timely modulation of the immune response can prevent disease progression and the fact that patients who are more likely to have worse outcomes from COVID-19, like patients with respiratory insufficiency, may also be the patients who benefit most from corticosteroid therapy.

Les et al. performed a randomized controlled trial in patients hospitalized due to COVID-19 without respiratory insufficiency after the first week of symptoms and with raised inflammatory markers (24). Patients were randomized to receive a 3-day course of intravenous methylprednisolone (120 mg/day) or placebo. The authors did not find differences among groups in preventing progression to respiratory failure or death during hospitalization.

Our study has several differences compared to this randomized controlled trial. The first is the larger sample size of our study. Analytical criteria to define inflammation were different, except for CRP; only one elevated marker instead of two was enough to be eligible. Although the patient profile was similar in both studies (young individuals with a low frequency of comorbidities), this study included patients from the second wave of COVID-19 before vaccine development and application. Consequently, the proportion of events was higher than ours (28% versus 14%). Additionally, a short course (3 days) of high-dose methylprednisolone was used instead of dexamethasone for seven days. Nevertheless, the outcome was similar regarding the efficacy of corticosteroids in preventing ARDS development.

There are potential side effects of corticosteroids, like hyperglycemia or bacterial infections. Our study showed that using a short course of dexamethasone in this group of patients may be safe. Previous studies showed similar results (25).

Our study has several limitations—first, the small sample size. We did not reach the targeted number of patients due to the low rate of ARDS development (14% instead of an expected incidence of 30%) and the end of the COVID-19 surge that emptied hospitals of COVID-19 patients. The trial may have been underpowered to identify significant differences. We found a low ARDS incidence compared to previous studies because our patients were young, without substantial comorbidities and, with one-third of patients vaccinated and with mild disease. Patients with inflammatory markers without respiratory insufficiency after seven days of infection may represent a subgroup with a good prognosis. However, the interim futility analysis was close to demonstrate non-significant differences among groups. The open design of the study may introduce biases, as both patients and healthcare providers were aware of the assigned interventions, which could influence treatment decisions and the reporting of outcomes.

In conclusion, among patients with COVID-19 pneumonia without oxygen needs but at risk of progressing to severe disease, early dexamethasone administration was safe but did not lead to a decrease in ARDS development.

## Data availability statement

The datasets presented in this article are not readily available because protection of personal data (organic law 3/2018 of 5 December on the protection of personal data and guarantees of digital rights and the general data protection regulation), Spain. Requests to access the datasets should be directed to anaisabel.franco@salud.madrid.org.

## Ethics statement

The studies involving humans were approved by Clínico San Carlos University Hospital. The studies were conducted in accordance with the local legislation and institutional requirements. The participants provided their written informed consent to participate in this study.

## Group members of EARLY-DEX COVID-19 research group

Belén Escolano-Fernández, Nuria Alfaro-Fernández, Mateo Balado-Rico, Ana Rocío Romero-Paternina, Esther Piniella-Ruiz, Ester Alonso-Monge, and Helena Notario-Leo, Internal Medicine Department, Hospital Universitario Infanta Leonor–Virgen de la Torre, Madrid, Spain; Carlos Bibiano-Guillén and Armando Antiqueira-Pérez, Emergency Department, Hospital Universitario Infanta Leonor–Virgen de la Torre, Madrid, Spain; Noemí Cabello-Clotet, Internal Medicine Department, Hospital Universitario Clínico San Carlos, Madrid, Spain.

## Author contributions

AF-M: Writing – review & editing, Writing – original draft, Visualization, Validation, Supervision, Software, Resources, Project administration, Methodology, Investigation, Funding acquisition, Formal analysis, Data curation, Conceptualization. MA-G: Writing – review & editing, Writing – original draft, Supervision, Investigation. MC-S: Writing – original draft, Investigation. NL-S: Writing – original draft, Investigation. MC-L: Writing – original draft, Investigation. FI-E: Writing – original draft, Investigation. CH-B: Writing – original draft, Investigation. JJ-T: Writing – original draft, Investigation. IV-M: Writing – original draft, Investigation. RR-P: Writing – review & editing, Writing – original draft, Investigation. GP-L: Writing – original draft, Investigation. IE-R: Writing – original draft, Supervision, Methodology. JT-M: Writing – review & editing, Writing – original draft, Visualization, Validation, Supervision, Software, Resources, Project administration, Methodology, Investigation, Funding acquisition, Formal analysis, Data curation, Conceptualization.
