# Supplementary material for: Effect of early administration of dexamethasone in patients with COVID-19 pneumonia without acute hypoxemic respiratory failure and risk of development of acute respiratory distress syndrome: EARLY-DEX COVID-19 trial
Source: Front Med (Lausanne). 2024 Jul 17;11:1385833. doi: 10.3389/fmed.2024.1385833 (PMC11290468; doi:10.3389/fmed.2024.1385833)
Supplement: Supplementary file 1 [file Data_Sheet_1.docx]

**Appendix A. Futility analysis**

To evaluate the possibility of continuing with the study, the following futility analysis was proposed, considering the hypotheses:

H0: There are no statistically significant differences between the treatments.

H1: There are statistically significant differences between the treatments.

If the null hypothesis is rejected, it is concluded that the treatments behave significantly differently, and differences could be achieved if the study continues.

If the null hypothesis is not rejected, it is concluded that the treatments behave similarly, so it would be futile to continue with it.

| **Design Information** | |
| --- | --- |
| **Statistic Distribution** | Normal |
| **Boundary Scale** | MLE |
| **Alternative Hypothesis** | Upper |
| **Early Stop** | Accept/Reject Null |
| **Method** | O'Brien-Fleming |
| **Boundary Key** | Both |
| **Alternative Reference** | 0.1 |
| **Number of Stages** | 4 |
| **Alpha** | 0.05 |
| **Beta** | 0.2 |
| **Power** | 0.8 |
| **Max Information (Percent of Fixed Sample)** | 114.0359 |
| **Max Information** | 705.0336 |
| **Null Ref ASN (Percent of Fixed Sample)** | 60.79268 |
| **Alt Ref ASN (Percent of Fixed Sample)** | 78.00977 |
| **Adj Design Alpha** | 0.05 |
| **Adj Design Beta** | 0.19992 |
| **Adj Design Power** | 0.80008 |
| **Adj Design Max Information (Percent of Fixed Sample)** | 114.069 |
| **Adj Design Max Information** | 705.4054 |
| **Adj Design Null Ref ASN (Percent of Fixed Sample)** | 60.76099 |
| **Adj Design Alt Ref ASN (Percent of Fixed Sample)** | 77.97327 |

MLE: Maximum Likelihood Estimation

| **Method Information** | | | | | | | | |
| --- | --- | --- | --- | --- | --- | --- | --- | --- |
| **Boundary** | **Method** | **Alpha** | **Beta** | **Unified Family** | | | **Alternative Reference** | **Drift** |
|  |  |  |  | **Rho** | **Tau** | **C** |  |  |
| Upper Alpha | O'Brien-Fleming | 0.05000 | . | 0.5 | 0 | 1.65589 | 0.1 | 2.655247 |
| Upper Beta | O'Brien-Fleming | . | 0.20000 | 0.5 | 0 | 0.99935 | 0.1 | 2.655247 |

| **Boundary Information (MLE Scale) Null Reference=0** | | | | | | |
| --- | --- | --- | --- | --- | --- | --- |
| **Stage** |  | | | **Alternative** | **Boundary Values** | |
|  | **Information Level** | | | **Reference** | **Upper** | |
|  | **Proportion** | **Actual** | **N** | **Upper** | **Beta** | **Alpha** |
| 1 | 0.2500 | 176.2584 | 130.4312 | 0.10000 | -0.05055 | 0.24945 |
| 2 | 0.5000 | 352.5168 | 260.8624 | 0.10000 | 0.02473 | 0.12473 |
| 3 | 0.7500 | 528.7752 | 391.2936 | 0.10000 | 0.04982 | 0.08315 |
| 4 | 1.0000 | 705.0336 | 521.7248 | 0.10000 | 0.06236 | 0.06236 |

MLE: Maximum Likelihood Estimation

| **Sample Size Summary** | |
| --- | --- |
| **Test** | Two-Sample Proportions |
| **Null Proportion** | 0.7 |
| **Proportion (Group A)** | 0.8 |
| **Test Statistic** | Z for Proportion |
| **Reference Proportions** | Alt Ref |
| **Max Sample Size** | 521.7248 |
| **Expected Sample Size (Null Ref)** | 253.1321 |
| **Expected Sample Size (Alt Ref)** | 356.9019 |

| **Sample Sizes (N) Two-Sample Z Test for Proportion Difference** | | | | | | | | |
| --- | --- | --- | --- | --- | --- | --- | --- | --- |
| **Stage** | **Fractional N** | | | | **Ceiling N** | | | |
|  | **N** | **N (Grp 1)** | **N (Grp 2)** | **Information** | **N** | **N (Grp 1)** | **N (Grp 2)** | **Information** |
| 1 | 130.43 | 65.22 | 65.22 | 176.3 | 132 | 66 | 66 | 178.4 |
| 2 | 260.86 | 130.43 | 130.43 | 352.5 | 262 | 131 | 131 | 354.1 |
| 3 | 391.29 | 195.65 | 195.65 | 528.8 | 392 | 196 | 196 | 529.7 |
| 4 | 521.72 | 260.86 | 260.86 | 705.0 | 522 | 261 | 261 | 705.4 |

| **Ceiling-Adjusted Design Boundary Information (MLE Scale) Null Reference=0** | | | | | | |
| --- | --- | --- | --- | --- | --- | --- |
| **Stage** |  | | | **Alternative** | **Boundary Values** | |
|  | **Information Level** | | | **Reference** | **Upper** | |
|  | **Proportion** | **Actual** | **N** | **Upper** | **Beta** | **Alpha** |
| 1 | 0.2529 | 178.3784 | 132 | 0.10000 | -0.04888 | 0.24658 |
| 2 | 0.5019 | 354.0541 | 262 | 0.10000 | 0.02499 | 0.12423 |
| 3 | 0.7510 | 529.7297 | 392 | 0.10000 | 0.04987 | 0.08303 |
| 4 | 1.0000 | 705.4054 | 522 | 0.10000 | 0.06235 | 0.06235 |

MLE: Maximum Likelihood Estimation

##

According to the previous results, Z-test values that are below -0.04888 result in a rejection of the null hypothesis, while values above 0.24658 accept the null hypothesis. If the statistic is outside the acceptance and rejection regions, the test should continue.

**Comparison of both groups**

If we analyze the information from both groups, the parameter estimate is 0.0284, and its standard error is 0.0658.

| **Obs** | **Parameter** | **Estimate** | **StdErr** | **Scale** | **Stage** |
| --- | --- | --- | --- | --- | --- |
| **1** | group | 0.0284 | 0.0658 | MLE | 1 |

MLE: Maximum Likelihood Estimation

| **Test Information (MLE Scale) Null Reference=0** | | | | | | | |
| --- | --- | --- | --- | --- | --- | --- | --- |
| **Stage** |  | | **Alternative** | **Boundary Values** | | **Test** | |
|  | **Information Level** | | **Reference** | **Upper** | | **group** | |
|  | **Proportion** | **Actual** | **Upper** | **Beta** | **Alpha** | **Estimate** | **Action** |
| 1 | 0.3276 | 231.0578 | 0.10000 | -0.01305 | 0.17860 | 0.02838 | Continue |
| 2 | 0.5019 | 354.0541 | 0.10000 | 0.02277 | 0.12726 | . |  |
| 3 | 0.7510 | 529.7297 | 0.10000 | 0.04981 | 0.08322 | . |  |
| 4 | 1.0000 | 705.4054 | 0.10000 | 0.06242 | 0.06242 | . |  |

MLE: Maximum Likelihood Estimation

**Test Plot for group**

**Conclusions**

Comparing Group A with Group B, differences could be found between groups A and B, although the results obtained indicate that they are close to entering the zone of non-rejection of the null hypothesis; that is, they are close to entering the zone of deciding to stop the study.
